# Draft Genome Sequence of the Deep-Subsurface Actinobacterium *Tessaracoccus lapidicaptus* IPBSL-7^T^

**DOI:** 10.1128/genomeA.01078-16

**Published:** 2016-09-29

**Authors:** Fernando Puente-Sánchez, Dietmar H. Pieper, Alejandro Arce-Rodríguez

**Affiliations:** a Systems Biology Program, Centro Nacional de Biotecnología, Consejo Superior de Investigaciones Científicas (CSIC), Madrid, Spain; b Microbial Interactions and Processes Research Group, Helmholtz Centre for Infection Research, Braunschweig, Germany

## Abstract

The type strain of *Tessaracoccus lapidicaptus* was isolated from the deep subsurface of the Iberian Pyrite Belt (southwest Spain). Here, we report its draft genome, consisting of 27 contigs with a ~3.1-Mb genome size. The annotation revealed 2,905 coding DNA sequences, 45 tRNA genes, and three rRNA genes.

## GENOME ANNOUNCEMENT

Members of the genus *Tessaracoccus* are Gram-positive, nonmotile, nonsporulating, and facultative anaerobic actinobacteria (1). Two species, *T. profundi* and *T. lapidicaptus*, have been isolated from deep-subsurface samples (2, 3). *T. lapidicaptus* was recently isolated from a 297-m-deep sample obtained from a drilling project that aimed to intersect the groundwater interacting with the massive pyrite minerals present in the Iberian Pyrite Belt (southwest Spain) subsurface (4). This environment is of special interest, as underground microbial oxidation of pyrite has been hypothesized to be responsible for the unique characteristics of Río Tinto (“Red River”), a natural extremely acidic stream with high concentrations of heavy metals (5). Furthermore, *T. lapidicaptus* possesses bioremediation potential due to its ability to mediate the biological precipitation of iron-rich phosphates and carbonates under anaerobic conditions (6). The study of its genome may help to identify genes involved in iron biomineralization and heavy metal resistance and to elucidate the particular adaptations that allow this microorganism to thrive under the extreme energetic and nutritional limitations that are characteristic of deep-subsurface environments (7).

A lyophilized sample of *T. lapidicaptus* IPBSL-7^T^ (DSM-27266) was obtained from the German Collection of Microorganisms and Cell Cultures (DSMZ, Braunschweig, Germany). Briefly, the cell pellet was rehydrated and cultured for 2 days in 5 ml of tryptic soy broth at 30°C. Total genomic DNA was isolated from 2 ml of the culture using the DNeasy blood and tissue kit (Qiagen, Düsseldorf, Germany). After DNA shearing (Covaris, Woburn, MA, USA), Illumina paired-end libraries were prepared using the NEBNext Ultra DNA library prep kit (NEB, Ipswich, MA, USA) and subjected to 250-bp paired-end sequencing on an Illumina MiSeq platform (Illumina, San Diego, CA, USA), which generated a total of 1,989,155 read pairs. Adaptor sequences were removed with Cutadapt version 1.10 (8), and reads were quality-trimmed by using PRINSEQ-lite version 0.20.4 (9). The resulting 1,245,382 pairs and 623,804 singletons were assembled with SPADES version 3.8.2 (10), and contigs that matched either the phiX174 genome or had less than 100× coverage were excluded. This yielded 27 contigs, with an *N*_50_ of 250,005 bases, a total length of 3,088,030 bases, an average coverage of 145×, and a GC content of 73%. Gene prediction and annotation were carried out using the RAST pipeline (11) as provided by the PATRIC server (12). A total of 2,905 coding DNA sequences, 45 tRNA genes, and three rRNA genes were identified.

### Accession number(s).

The nucleotide sequence and annotation data for the *Tessaracoccus lapidicaptus* IPBSL-7^T^ draft genome have been deposited at DDBJ/ENA/GenBank under the accession number MBQD00000000. The version described in this paper is the first version, MBQD01000000.
